# Differential RhoA Dynamics in Migratory and Stationary Cells Measured by FRET and Automated Image Analysis

**DOI:** 10.1371/journal.pone.0004082

**Published:** 2008-12-30

**Authors:** John Paul Eichorst, Shaoying Lu, Jing Xu, Yingxiao Wang

**Affiliations:** 1 Center of Biophysics and Computational Biology, University of Illinois at Urbana-Champaign, Urbana, Illinois, United States of America; 2 Department of Bioengineering, University of Illinois at Urbana-Champaign, Urbana, Illinois, United States of America; 3 Beckman Institute for Advanced Science and Technology, Department of Molecular and Integrative Physiology and Neuroscience Program, University of Illinois at Urbana-Champaign, Urbana, Illinois, United States of America; Massachusetts General Hospital/Harvard University, United States of America

## Abstract

Genetically-encoded biosensors based on fluorescence resonance energy transfer (FRET) have been widely applied to study the spatiotemporal regulation of molecular activity in live cells with high resolution. The efficient and accurate quantification of the large amount of imaging data from these single-cell FRET measurements demands robust and automated data analysis. However, the nonlinear movement of live cells presents tremendous challenge for this task. Based on image registration of the single-cell movement, we have developed automated image analysis methods to track and quantify the FRET signals within user-defined subcellular regions. In addition, the subcellular pixels were classified according to their associated FRET signals and the dynamics of the clusters analyzed. The results revealed that the EGF-induced reduction of RhoA activity in migratory HeLa cells is significantly less than that in stationary cells. Furthermore, the RhoA activity is polarized in the migratory cells, with the gradient of polarity oriented toward the opposite direction of cell migration. In contrast, there is a lack of consistent preference in RhoA polarity among stationary cells. Therefore, our image analysis methods can provide powerful tools for high-throughput and systematic investigation of the spatiotemporal molecular activities in regulating functions of live cells with their shapes and positions continuously changing in time.

## Introduction

Cell migration plays important roles in embryonic development, tissue repair, cancer invasion and atherosclerosis [1]. It is a highly integrated process modulated by multiple signaling pathways, consisting of cytoskeleton extension and focal complex formation at the front, and focal adhesion disassembly and cytoskeleton contraction at the back [1]. The family of small Rho GTPases plays key roles in regulating actin cytoskeleton and hence migration [1]–[3]. These GTPases function as molecular switches in the cell by alternating between active GTP-bound and inactive GDP-bound states, mediated by guanine nucleotide exchange factors (GEFs) and GTPase activating proteins (GAPs), respectively [1]–[4]. Among the prominent members of small Rho GTPases, such as RhoA, Rac1, and Cdc42 [2], [3], RhoA was reported to mediate the assembly of contractile actomyosin filaments at the rear of migrating cells via one of its downstream effectors, ROCK [3]–[5]. Therefore, it has been suggested that RhoA activity may concentrate at the tail of migrating cells [3]. However, by using biosensors based on fluorescence resonant energy transfer (FRET) [6], a surprisingly high RhoA activity has recently been observed to present not only at the tail but also at the front of migrating cells [7], [8]. These results suggest that RhoA may have different functions at different subcellular locations.

FRET is a phenomenon of quantum mechanics. When two fluorescent proteins (FPs), a donor and an acceptor, are in proximity with favorable orientation, the excitation of donor FP can induce the emission from the acceptor [9]. FRET has enabled the development of a variety of biosensors capable of detecting molecular activities or protein-protein interactions in live cells [10], [11]. Two FRET-based RhoA biosensors have been developed to visualize spatiotemporal dynamics of RhoA activities in live cells [7], [12]. Matsuda's group developed a RhoA biosensor (Raichu-RhoA) which contains RhoA (amino acid 1–189), a flexible linker, and the RhoA-binding (RBD) domain from its substrate molecule PKN, concatenated in between a cyan and a yellow fluorescent protein (CFP and YFP) [12]. The activation of RhoA can induce the intramolecular binding between RhoA and the RBD domain in the biosensor. The resulting conformational change can bring the CFP and YFP into close proximity and enhance FRET signals. The Raichu-RhoA biosensor has enabled the monitoring of RhoA activity during cell division, polarization and focal adhesion turnover in different cells [8], [12]–[15]. Another FRET RhoA biosensor was independently developed, containing sequentially a full-length RhoA, CFP, a flexible linker, YFP, and the RBD domain of rhotekin [7]. This biosensor has also been successfully applied to study various cellular functions, including migration and polarity during chemotaxis [7], [16], [17].

While the development of the FRET biosensors has greatly advanced our knowledge of signaling transduction in live cells, the vast amount of imaging data produced by these biosensors requires automatic, intelligent, and objective image analysis tools to allow precise and efficient interpretation of biological information [18]. In particular, efficient algorithms are in great need to track the cell movement and to account for the difference in the shape and geometry of individual cells.

Image registration has been widely applied in engineering and science for automatically tracking moving objects in time and for finding the pixel-wise correspondence between two images of similar objects [19], [20]. In live-cell image analysis, this method has been used to align, via simple translation and rotation, the fluorescent images at different wavelengths [21]–[23] and the nucleus images at different time points of the same cell [24]. It has also been applied to track fluorescent particles inside the cells [25]. More sophisticated image registration methods have also been applied to analyze in situ gene expression in the mouse brain [20]. Other quantification methods have been developed to study the distribution of protein and the polarity of molecular activity by dividing the whole cell into small wedges in the polar coordinated system [23], [26]–[28], to study the motility and energy of single sperms by tracking and trapping [29], [30], or to extract data on 3D vascular anatomy [31]. However, automated whole-cell image registration of moving cells by pixel-wise tracking is still in great need for the high-resolution and efficient quantification of molecular signals in space and time.

In this paper, we have developed automated image analysis methods to track the movement of single cells and quantify the FRET signals at subcellular regions utilizing the RhoA biosensor from Dr. Matsuda [12]. The spatiotemporal patterns of RhoA activation were analyzed in migratory and stationary HeLa cells in response to growth factor stimulation. Our results suggest that the global RhoA activity in migratory cells was down-regulated in response to growth factor stimulation to a lesser extent than that in stationary cells. The RhoA activity was more concentrated in the opposite direction of migration in migratory cells, but did not show preference in stationary cells.

## Materials and Methods

### Cell Lines and Culture

HeLa cells (ATCC, Manassas, Virginia) were cultured in a humidified 95% air, 5% CO_2_ incubator at 37°C. The culture medium was Dulbecco's modified Eagle's medium (DMEM) supplemented with 10% fetal bovine serum, 2 mM L-glutamine, 1 unit/ml penicillin, 100 µg/ml streptomycin, and 1 mM sodium pyruvate. The cell culture reagents were obtained from Invitrogen (San Diego, California).

### Transient Transfection and Microscope Imaging

Since the Raichu-RhoA biosensor can be easily transfected and is well-characterized for HeLa cells, we used this biosensor to visualize the dynamics of RhoA activity in HeLa cells on fibronectin covered glass [12]. The Raichu-RhoA biosensor was transfected into HeLa using Lipofectamine (Invitrogen) [12] before starvation with 0.5% FBS for 36–48 hours. The cells were then suspended in trypsin-EDTA and seeded on fibronectin-coated glass-bottom dishes for 3–6 hours before EGF (50 ng/ml) stimulation. During imaging, the cells were kept in CO_2_-independent medium without serum (Invitrogen) at 37°C. The objective focus was aimed near the basal side of the cell. Images were collected by a Zeiss axiovert inverted microscope with a 420DF20 excitation filter, a 450DRLP dichroic mirror, two emission filters controlled by a filter changer (480DF30 for CFP and 535DF25 for YFP), and a cooled charge-coupled device camera (Cascade 512B; Photometrics). The intensity of excitation light was controlled by adjustable neutral density filters to ensure minimal photobleaching during imaging. The CFP and YFP fluorescence intensity images were background subtracted. The FRET ratio images were computed by calculating the pixel-wise CFP/YFP ratio to represent RhoA activity in space and time, using MetaFluor 6.2 software (Universal Imaging) or our customized programs developed in MATLAB (MathWorks).

### Image Registration

**Figure 1:**
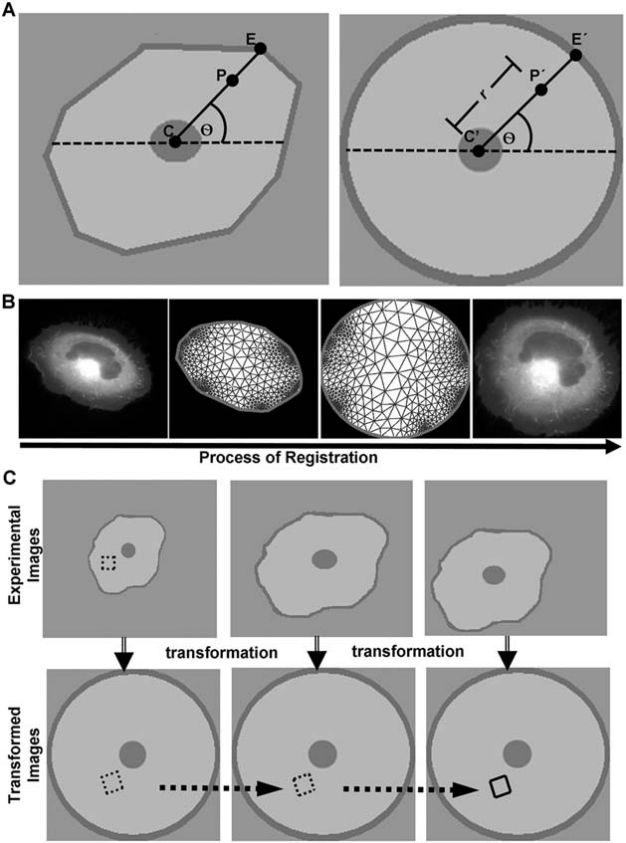
The diagrams depicting the region-tracking algorithm. (A) The centroid of the cell (*C*), a control point (*P*), and the boundary intersection at the convex hull (*E*, in left image) were used to compute the relative distance and orientation of the vector pointing from the centroid to the control point. (*r*,*θ*) is the polar coordinate of the point on the unit disk (*P′*) corresponding to the control point (*P*) in the cell image (right image). (B) The registration process is shown from left to right. First, the cell is partitioned into a triangular mesh within its boundary. Second, the mesh was mapped to a unit circle by computing the relative distance and orientation of the vectors pointing from the centroid to mesh nodes (or control points). Finally, the control points and cell image were mapped to the unit disk by a piecewise linear interpolation within the triangular mesh. (C) The top panels show the images of a single cell undergoing expansion or translocation, with a selected ROI in the first frame. The lower panels show the unit disks transformed from the cell at different time points. The boundary of the transformed ROI in the first frame is used to track and quantify the signals from the same region in these different unit disks.

To automatically register the time-lapse images of a moving cell, we mapped these cell images into a unit disk, which served as a reference image both for the registration of the time-lapse image sequence of the same cell and for the comparison between different cells with varying shapes. It took four steps to map a cell to the unit disk. First, the cell body was determined by segmentation. Second, the control points on the cell were computed by Delaunay triangulation of the convex hull of the cell body. Third, the control points were mapped to the reference image (unit disk) by calculating the relative position of the points in the convex hull. Finally, the cells were mapped into the unit disk by a piecewise linear transformation [32] defined by the control points and the Delaunay triangulation (Figure 1).

Otsu's method [33] was used to compute a threshold of intensity for the segmentation of the cell body, because the fluorescent images recorded using the RhoA biosensor were bright and readily separable from the background. A list of nodes outlining the contour of the cell was generated based on the identified cell body. Inside the contour, a mask image was hence created to represent the shape of the cell. The images collected from the YFP channel were used for the detection of cell edge and the mask because they were brighter than those from the CFP channel.

To register the shape of a cell by a unit disk without ambiguity, it is required that the cell shape be convex. Therefore, the convex hull circumscribing the cell mask image was calculated [34] and used as the boundary for computing the Delaunay triangulation [35]. The nodes in the triangulation were then used as the control points for the cell image. For each control point in the cell image, the corresponding control point in the unit disk was calculated based on its relative position in the convex hull. In brief, the centroid of the cell (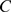) was mapped to the center of the unit disk (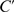) (Figure 1A). For any other control point (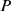), the corresponding point on the unit disk, 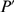, was calculated based on the relative position of the point 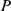 between the centroid and the edge of the convex hull. Suppose 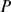 has the polar coordinates of (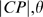), centered at the centroid 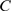; and the ray from 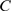 to 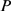 crosses the edge of the convex hull at the point 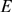. Then the polar coordinates of the corresponding point (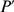) in the unit disk is given by (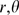) centered at 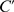, where 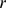 represents the relative distance from 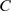 to 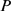, i.e. 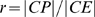 (Figure 1A–B). Conversely, any point in the unit disk, 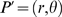, can be mapped back onto different cells or the same cell with different shapes at different time points.

The convex hull of the whole cell was mapped onto the unit disk by a piecewise linear transformation [32]. Within each triangle in the Delaunay triangulation, the transformation is linear and uniquely defined by the three control points which are nodes of the triangle (Figure 1B). The transformation is also continuous at the edges shared by two triangles.

### Tracking the Regions of Interest (ROIs)

To track the regions of interest, the time-lapse images of a single cell were first transformed into a series of unit disks. One or multiple ROIs were then selected and defined by the user on the first cell image. Thereafter, a mask for the ROI was generated and mapped to the first unit disk by our image registration method. To track the average FRET signal in this region, the mask of ROI in the unit disk was assumed to remain at the same location in time (Figure 1C). Therefore, the same mask can be applied to all the unit disk images to track and calculate the time course of average FRET ratio on the selected region(s), which was computed by taking the ratio of the total CFP and YFP intensity within the region(s) in the cell (Figure 1C). The assumption of constant ROIs in the reference unit disks is accurate if the cells only translate in the imaging plane, or shrink/expand in the radial directions extending from the centroid. The cells were not expected to rotate around their centroids during the course of experiment. These assumptions are reasonable for slow migration cells within the time window of our experiments and consistent with the graded radial extension (GRE) model [36], where the cells were expected to extend or retract their lamellipodia locally perpendicular to the cell edge and along the actin fibers.

The ROI(s) on the reference unit disk was mapped back to the original cell images to visually confirm that they can indeed track the actual region in migrating cells (Fig. 1C). To improve computational efficiency, only the nodes outlining the ROI on the unit disk, but not the whole mask, were transformed back onto the cell images.

### Cluster Analysis

To examine the geometric features of subcellular regions with similar levels of RhoA activity, the pixels of the cell image were classified and divided into six clusters according to their RhoA FRET ratio values. The MATLAB function *kmeans* was used to classify the pixels into different clusters, by minimizing the within-cluster variation of the FRET values [37], [38]. As a result, each cluster contained pixels with similar FRET ratio values.

The MATLAB programs implementing these functions can be obtained by writing to the corresponding author of this paper.

## Results

### Image Registration

Two classes of HeLa cells with significantly distinctive migration patterns were observed during experiments: migratory and stationary. To determine whether a HeLa cell was migratory or stationary, the location of its centroid was monitored in time. The furthest distance traveled by the centroid from the initial position was divided by the sum of the distances traveled at each time step for about 60 minutes in each experiment. With a ratio greater or equal to 0.5, a cell was considered to be migratory. Otherwise, the cell was considered stationary. The results confirmed that there are clearly two classes of HeLa cells with the distance ratios at 0.71 and 0.1867 (Table 1). The migration direction of each migrating cell can be determined from the trajectory of its centroid by linear fitting.

**Table 1:** Categorization of migrating and stationary cells.

Cell Type	Longest Distance_________________Total Displacement	Standard Error
Migrating Cells	0.71	0.03
Stationary Cells	0.1867	0.03266

The cells were categorized as migrating and stationary according to the ratio between the longest distance traveled and the total displacement of the cell during the experiments. This ratio is shown with standard error in the table.

**Figure 2:**
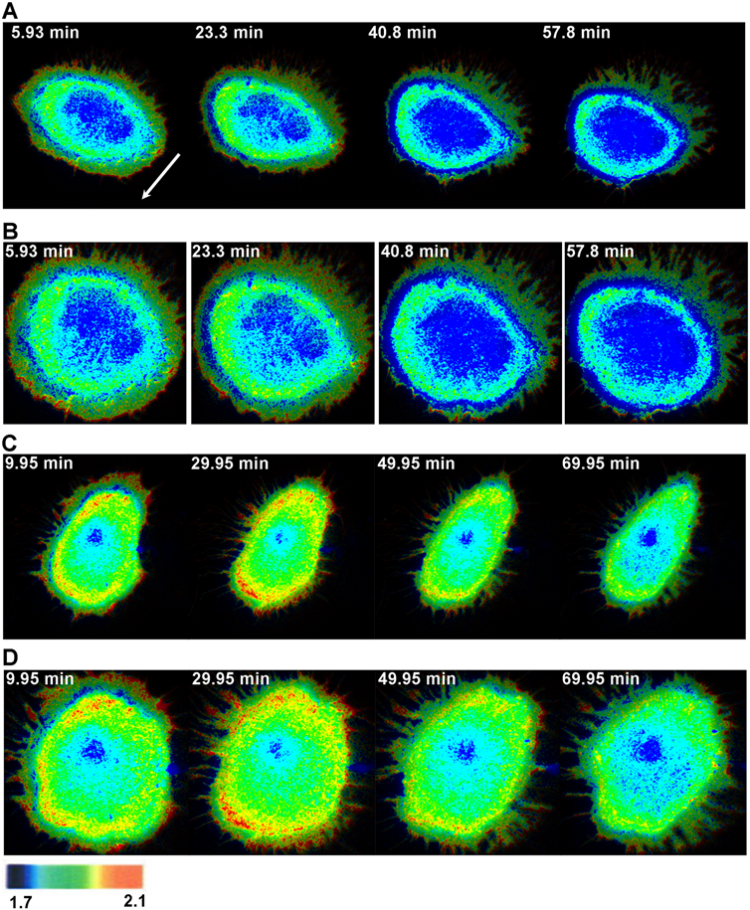
FRET images and their transformed maps on unit disks of the Raichu-RhoA biosensor (Venus/ECFP) in migrating and stationary cells. Panel (A) shows a sequence of time-lapse FRET images for a representative migrating HeLa cell. The white arrow points to the migration direction. Panel (B) shows the FRET images of the migrating cell in (A) transformed into a unit disk. The spatial temporal dynamics of the FRET images of the moving HeLa cell is also shown in Movie S1. Panel (C) shows a sequence of time-lapse FRET images for a representative stationary HeLa cell. Panel (D) shows the FRET images of the stationary cell in (B) transformed into a unit disk.

With our method of image registration, the intracellular RhoA activity under growth factor stimulation can be visualized within a uniform reference image frame (Figure 2). The RhoA activity was high at both the front and back edge of migrating cells (Figures 2A–2B). This confirmed that RhoA contributes to the actin filament elongation and the membrane ruffle at the cell front via mDia whereas regulates actomyosin contractility at the cell rear [2]. In both migratory and stationary cells, spatial patterns of two concentric ring-like subcellular structures with high RhoA activity can be clearly observed (Figure 2 and Movie S1). The outer ring with high RhoA activity may represent the nascent and dynamic integrin activation on RhoA activity [39]. The high RhoA activity in the inner ring may indicate the relatively high stress occurring at the convergent zone where retrograde actin flow of the lamella meets the anterograde actin flow of the cell body [40]. Despite these distinctive subcellular features, the overall RhoA activity of both migratory and non-migratory HeLa cells decreased after the EGF stimulation (Figure 2 and Movie S1). It appears that, upon EGF stimulation, the RhoA activity remained relatively stable at the back of the migratory, but not stationary cells (Figure 2B and Movie S1).

### Tracking the Regions of Interest

**Figure 3:**
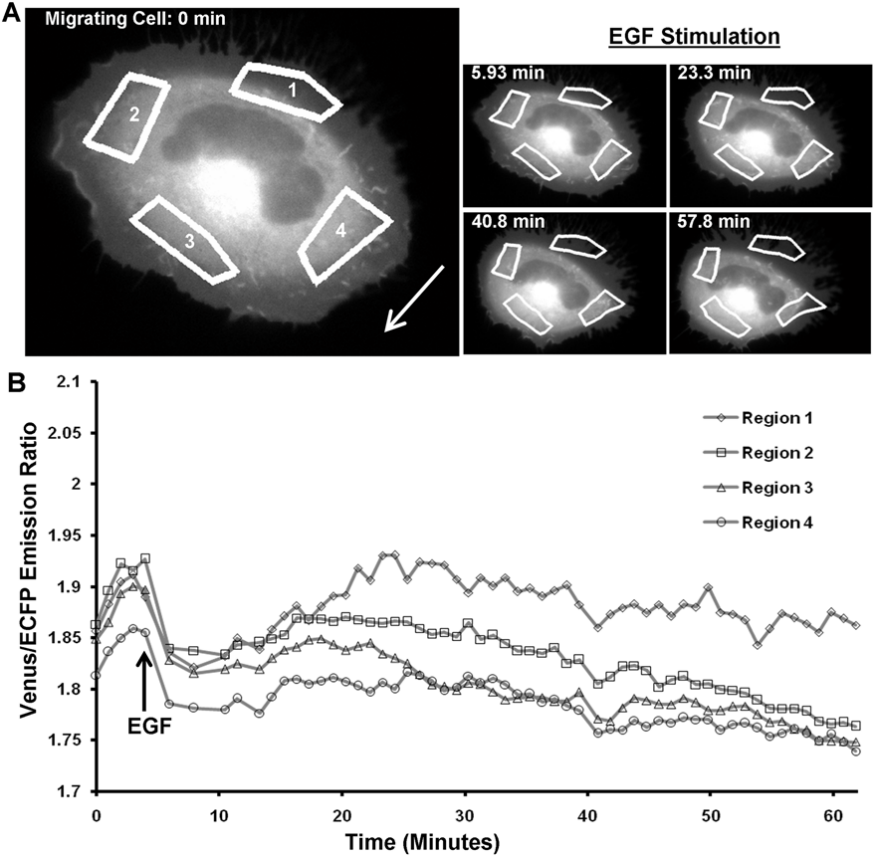
The tracking and analysis of the RhoA activity in different ROIs of a migrating HeLa cell. (A) Left: Four ROIs were chosen in the first frame to monitor the RhoA activity at the front, back, and two sides of a migrating cell. Right: The computed contours and locations of the ROIs in the cell at different time points based on our tracking algorithm. The time-lapse images of the automatically tracked ROIs are also shown in Movie S2. (B) The time courses of FRET ratios of the Raichu-RhoA biosensor averaged on the four ROIs shown in (A).

To track and quantify the local RhoA activities at subcellular regions in HeLa cells, four ROIs were selected in both the migrating and stationary cells. The four ROIs in the migrating cells span the front, back and two sides of the cell (Figure 3A and Movie S2). Since a ROI may move out of the cell body (Figure 3A, Region 1), a threshold value of the YFP image of the cell was used to judge and calculate the FRET signal within the part of the ROI inside the cell body. Therefore, the average FRET ratios within each ROI of the migrating cells at different time points were calculated to monitor the dynamic RhoA activity at subcellular locations (Figure 3B). It appeared that the RhoA activity in all four regions immediately dropped and slightly recovered after EGF stimulation. The local RhoA activity then decreased continuously in regions at the front and both sides. However, in the region near the trailing edge of the migrating cell, the RhoA activity remained relatively constant without significant down-regulation after EGF stimulation (Figure 3B). This is a representative feature among different migrating cells analyzed. As a result, the difference between the activity of the ROIs near the front and the back of the cell displayed a significant increase for the migrating cells. This finding corroborates the previous study that high RhoA activities are important in mediating the tail contraction of migrating cells [2].

**Figure 4:**
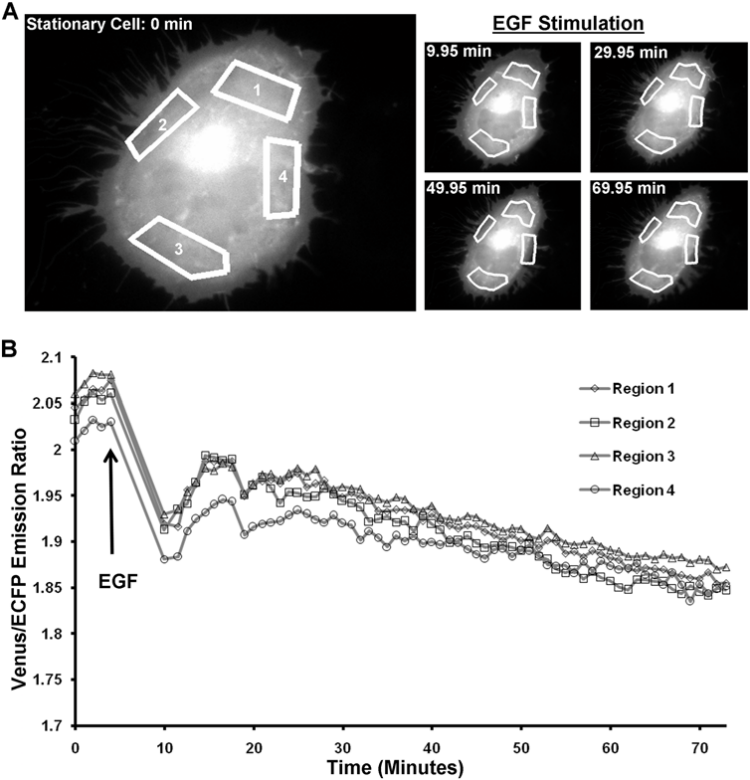
The tracking and analysis of the RhoA activity in different ROIs of a stationary HeLa cell. (A) Left: Four ROIs were chosen in the first frame to monitor the RhoA activity at the front, back, and two sides of a stationary cell. Right: The computed contours and locations of the ROIs in the cell at different time points based on our tracking algorithm. (B) The time courses of FRET ratios of the Raichu-RhoA biosensor averaged on the four ROIs shown in (A).

In the stationary cells, because the cells were not moving significantly, the positions of ROIs remained relatively unchanged in time (Figure 4A). The RhoA activity in all four regions immediately dropped and slightly recovered before continuing decrease after EGF stimulation. In contrast to the sub-cellular differences of RhoA activity observed in the migrating cells, the RhoA activity in all four ROIs of the stationary cells appeared to decrease with similar kinetics upon EGF stimulation (Figure 4).

**Figure 5:**
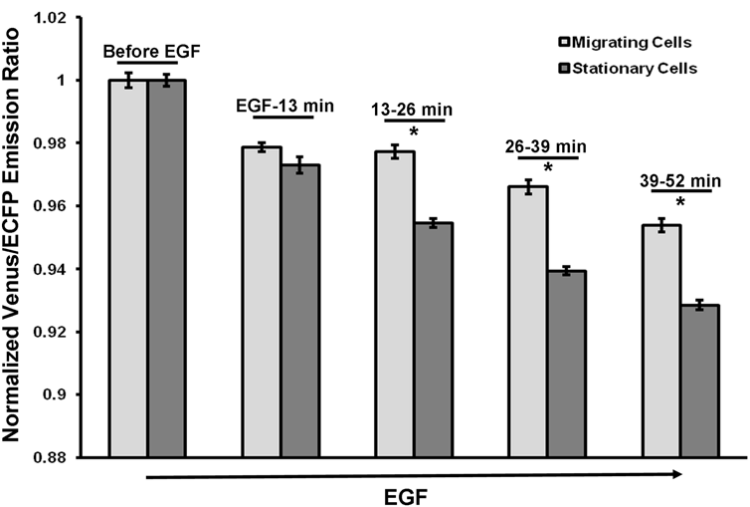
The global RhoA activity in migrating and stationary HeLa cells at different time points before and after EGF stimulation. The RhoA activity averaged over four ROIs spanning the whole cell body was used to represent the global RhoA activity. The error bars represent the standard error and the asterisks mark the time points where the average RhoA activities were significantly different (t-test, p<0.05) between migrating and stationary cells.

Due to the relatively stable RhoA activity at the tail of the migrating cells upon EGF stimulation, the global activity of RhoA in migrating cells was expected to decrease slower than that of the stationary cells. Hence we quantified the global activity of the RhoA biosensor in the migrating and stationary cells by taking the average of RhoA activities in all four ROIs and normalized according to their pre-EGF ratios. The results confirmed that the RhoA activity decreased in both the migrating and the stationary cells upon EGF, at a slower rate in migrating cells than that in stationary cells (Figure 5). The difference between the normalized ratios of the RhoA biosensor in migrating and stationary cells became statistically significant at 13–26 minutes after EGF stimulation (Figure 5), consistent with the time when the RhoA activity at the tail of the migrating cells started to show difference from other subcellular regions (∼25 minutes after EGF in Figure 3B). This difference between migrating and stationary cells continued to increase in time after EGF stimulation (Figure 5). These results indicate that the RhoA activity in migrating cells upon EGF stimulation is differentially down-regulated from that in stationary cells, possibly due to the relatively high RhoA activity at the back of migrating cells after EGF stimulation.

### Cluster Analysis

**Figure 6:**
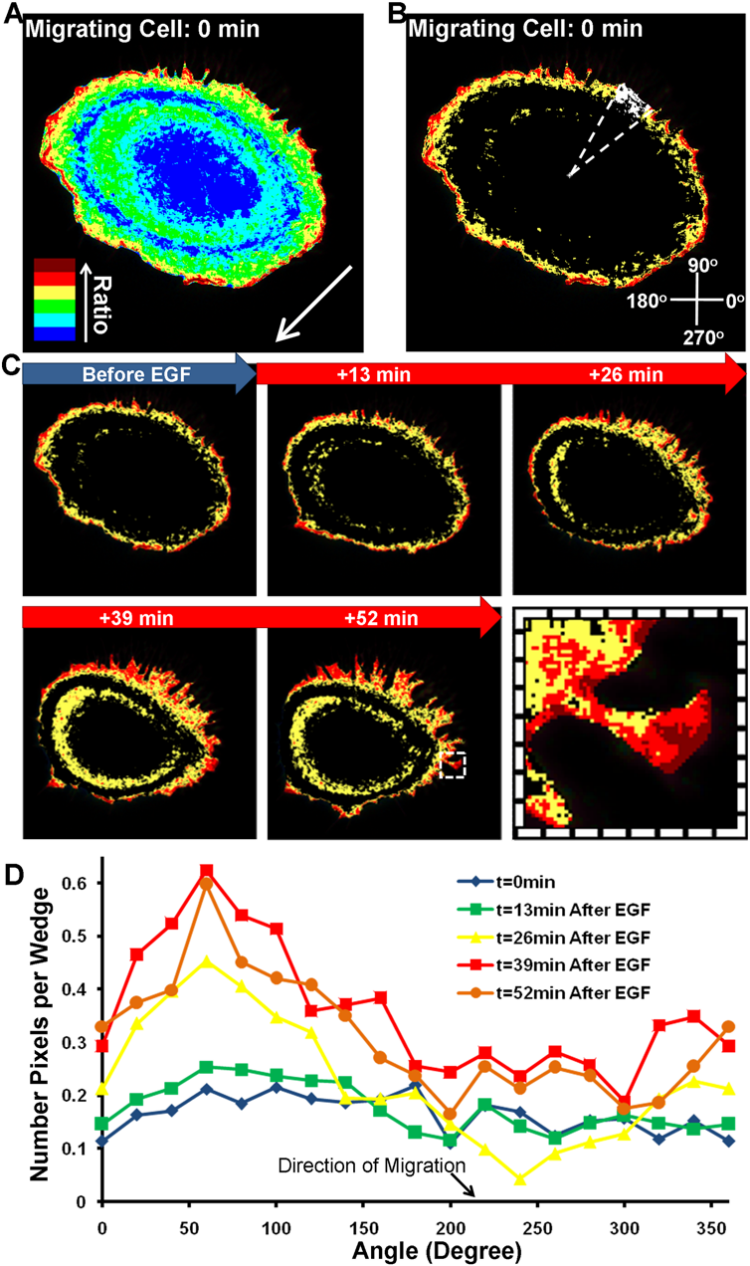
The cluster analysis of RhoA activity in a representative migrating cell. (A) The six subcellular regions with different RhoA activities were computed using the K-means cluster analysis. These regions are color-coded according to their RhoA activities, with cold and hot colors indicating low and high levels of RhoA activity, respectively. The migrating direction is indicated by the white arrow. (B) The three regions with the highest RhoA FRET ratios from panel (A) are selected and demonstrated. The white dotted lines outline a wedge in the cell. The coordinate system used for the polar analysis is shown in the lower right corner. (C) Time-lapse images showing the three clusters with high FRET ratio before and after EGF stimulation. The inset highlights the position of the cluster with the highest FRET ratio, which is typically located at the very edge of the cell. (D) The cell was divided into 18 wedges, as shown in panel (B). The number of pixels within the three clusters of highest FRET ratio in each wedge was normalized by dividing the total pixel number of the wedge. This normalized number of high-FRET pixels per wedge is plotted versus the angle of the wedge. The dynamic behavior of the automatically detected clusters is also shown in Movie S3.

We further studied the geometric features of the subcellular regions with relatively high RhoA activity after EGF stimulation. The intracellular pixels in the FRET image was divided into six clusters according to their FRET ratio values. As shown in Figure 6A, the pixels in the image were color-coded by the cluster they belong to, ordered from blue to red according to the mean RhoA FRET ratio in each cluster ranging from low to high. The top three clusters with the highest FRET ratio were selected and monitored to examine the distribution of high RhoA activity and their effect on cell behaviors, e.g. migration (Figures 6 and Movie S3). A MATLAB program was developed to select and quantify the area of these regions automatically (Figure 6). The results revealed that the area size of these selected regions increased in time after EGF stimulation, particularly so at the back of the migrating cells (Figure 6C). To quantitatively analyze the size of these regions with high RhoA activities, a polar coordinate system was established, centered at the centroid of each cell. The cell was then divided into 18 angular wedges with 20° increments (Figure 6B). The number of pixels in the entire wedge, 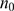, was calculated as well as the number of pixels within the three high-RhoA clusters in the wedge, 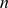. The normalized number of pixels in each wedge was calculated by taking the ratio of 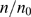. The normalized number of pixels in each wedge was plotted against the angle of the wedge to represent the polarity of RhoA activity. As shown in Figure 6D, this polarity curve of a representative migrating HeLa cell at different time points clearly demonstrates an increased spatial polarity of RhoA initiated by EGF stimulation. The pixels with relatively high RhoA activity appeared to accumulate in the region opposite to the migration direction, approximately at 220° in coordinates in Figure 6B.

**Figure 7:**
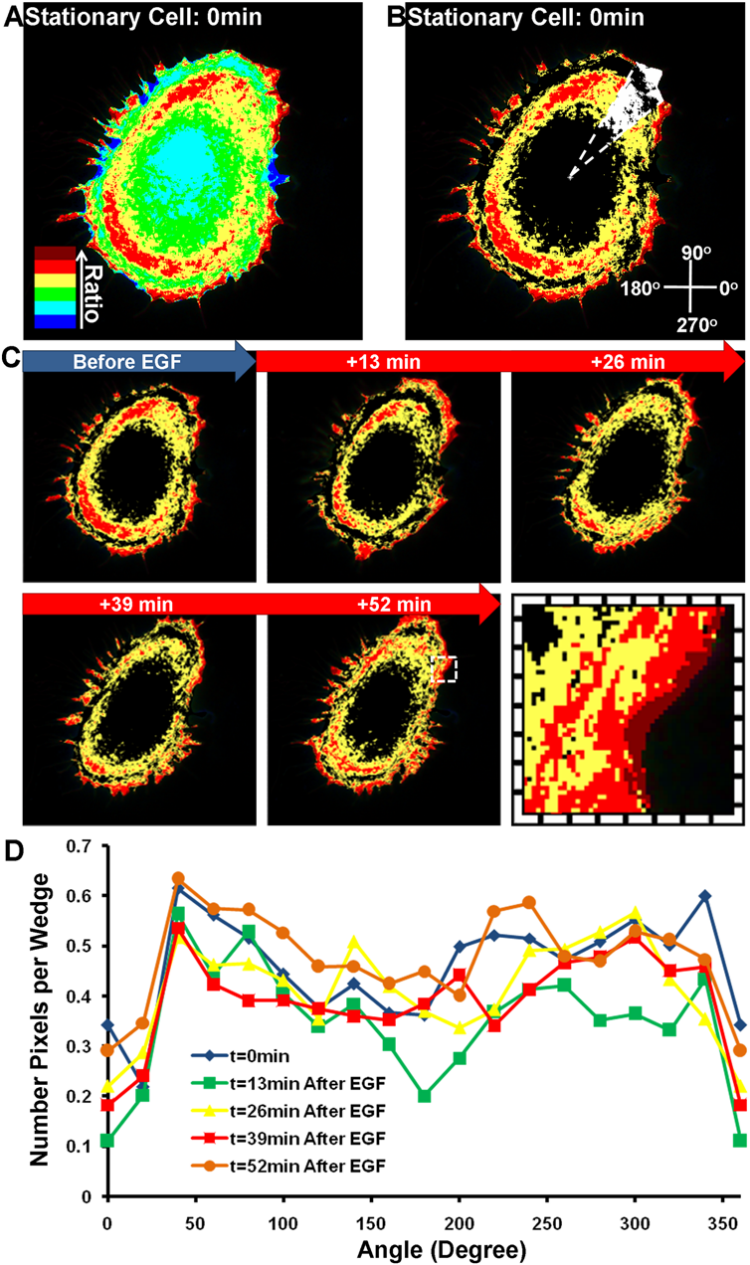
The cluster analysis of RhoA activity in a representative stationary cell. All the labeling and description are the same as in Figure 6, except that there is a lack of migration direction available for these stationary cells.

A similar cluster analysis was conducted on stationary cells. A representative stationary cell with six clusters is color-coded by the FRET ratio and depicted in Figure 7A, with the highest three clusters and their time-lapse images shown in Figures 7B and 7C, respectively. The normalized number of wedge pixels at different angles was quantified and plotted against angle in Figure 7D. The result indicates that there was no significant polarization process upon EGF stimulation in stationary cells.

**Figure 8:**
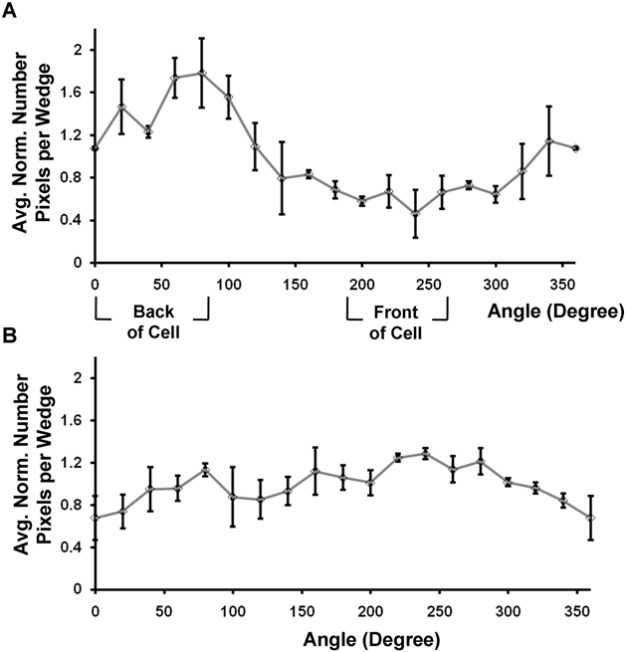
A comparison of the subcellular distribution of RhoA activity between the stationary and migrating cells after EGF stimulation. The normalized number of high-FRET pixels in each wedge is plotted against the angle of the wedge for multiple (A) migrating and (B) stationary cells at 52 minutes after EGF stimulation. The error bars represent standard error values. The migrating cells were rotated and aligned so that they had the same migration direction. The front and back of the migration direction were determined by the relative locations of the wedges in regard to their corresponding centroids.

The averaged polarity results from multiple migrating cells were calculated by aligning their direction of motion toward the same angle. At 52 minutes after EGF, the normalized number of pixels with high RhoA activity in each angular wedge clearly displayed a polarized pattern, with more pixels accumulating at the back of the migrating cells (Figure 8A). In contrast, the distribution of the regions with higher RhoA activity appeared more random in stationary cells (Figure 8B). All these results suggest that the polarization of RhoA activity in HeLa cells plays important roles in regulating the cell migration and its directions.

## Discussion

The recent advancement of live-cell imaging technologies has provided a variety of powerful tools and allowed the collection of a vast amount of image data/movies which requires significant time and effort on image analysis and quantification. However, there is a lack of user-friendly and automated image-analysis tools with high throughput capability. We have developed image analysis algorithms and methods based on the image registration for tracking the complex movement of migrating cells. These methods were applied to automatically track and quantify the time-lapse FRET ratio of Raichu-RhoA biosensor at subcellular regions in live HeLa cells, and to analyze the polarity of intracellular regions with high RhoA activities. The results suggest that the subcellular distribution of high RhoA activity is differentially regulated in migrating and stationary cells. These methods can also be applied to analyze the general fluorescence images of signaling transduction in live cells and enable an automated, efficient and objective quantification of a large quantity of imaging data.

The image analysis methods presented in this paper are completely automated. Because the ROIs were automatically tracked in moving cells, users only need to define the initial regions of interest in the first cell image. Each frame with four ROIs requires several seconds to quantify the FRET ratio with a personal computer. The automation of this kind of analysis is crucial because, at the current stage, the majority of data analysis for FRET imaging in live cells was performed manually with the contours of ROIs moved by a biologist if the cell moves or changes the shape. This manual tracking of ROIs for live cells can result in low-efficiency, tediousness, and inaccuracy. With our analysis method, the tracking is very reliable in general if the ROIs were selected inside the cell body and away from the cell edge. Because the convex hull or contour of the cell, but not the actual cell edge, was mapped to the unit disk, this method would become less accurate if a ROI was close to some cell edges which change their shapes between concave and convex. Further improvement of the automated tracking method would require the development and implementation of more sophisticated image registration algorithm. Nevertheless, the conversion of different cells into a uniform scheme, such as a unit disk, should be useful and important to standardize and compare the signals from each individual cell.

The application of the novel cluster analysis method is useful for analyzing and quantifying the subcellular distribution and the polarity of RhoA activity. The absolute value of RhoA activity did not present an obvious pattern of polarity. However, there was a significantly increase in the number of pixels with relatively higher RhoA activity at the tail of migrating cells upon EGF stimulation, evidenced by the quantification using the automated cluster analysis (Figure 6D). This highly-coordinated distribution of RhoA activity in space can likely contribute and control the direction of cell migration. The concentrated accumulation of high RhoA activity at the cell rear can help the detachment of cell tail from the substrate during migrating [41], possibly through the activation of downstream molecule ROCK which controls the phosphorylation of myosin light chain kinase (MLCK) and phosphatase (MLCP) to regulate the actomyosin contractility [5]. The inhibited RhoA activity at cell migration direction can lead to the activation of Rac1 [42], a small GTPase controlling cell protrusion and lamellipodia formation [43]. As such, cells can be coordinately guided to migrate toward the direction opposite to the RhoA polarity.

Comparing the results from migrating and stationary cells revealed distinct spatial patterns of RhoA activity in these two kinds of cells. In contrast to the polarized RhoA activity in migrating cells, the global down-regulation of the RhoA activity in stationary cells upon EGF stimulation may promote the protrusion in all directions without discrimination, which prevents a persistent and directional migration. The global change of RhoA activity upon EGF stimulation in migrating cells appeared less significant as compared to that in stationary cells. This is largely due to the consistent maintenance of a high RhoA activity at the trailing edge of migrating cells, but not in stationary cells. Further studies are needed to elucidate the detailed molecular mechanism governing the differential RhoA responses of stationary and migrating cells.

In summary, our image analysis method can allow a high-throughput and automated means for quantifying and analyzing the spatiotemporal molecular signals in live cells. This is particularly powerful in analyzing a large quantity of signals from cells with changing shapes, e.g. migrating cells. We have further demonstrated the significance of this method by applying it to quantify the dynamic RhoA activity at subcellular levels in migrating and stationary HeLa cells. The results revealed that EGF induced a down-regulation of RhoA in both migrating and stationary HeLa cells. However, a polarized distribution of RhoA activity can be consistently observed only in migrating, but not stationary cells.

## Supporting Information

Movie S1 The FRET images were mapped to a disk. The spatiotemporal dynamics of the FRET ratio representing RhoA activity upon EGF stimulation is shown in a typical migrating cell (left) and reference images mapped into a disk (right). Red and blue colors indicate high and low RhoA activity, respectively. The cell was shown to migrate in the view field, especially during the later part of the video (left), while the reference images of the cell remained within the static unit disk (right).
(9.81 MB MOV)

Movie S2 Four regions of interest were tracked in a migrating cell. The regions moved and changed shape as the cell migrated forward.
(7.93 MB MOV)

Movie S3 The geometric features of the six clusters (right) and three top clusters (left) in a migrating cell are shown in time. These regions are color-coded according to their RhoA activities, with cold and hot colors representing regions with low and high levels of RhoA activity, respectively.
(10.26 MB MOV)
